# Estimating foraging behavior in rodents using a modified paradigm measuring threat imminence dynamics

**DOI:** 10.1016/j.ynstr.2023.100585

**Published:** 2023-11-07

**Authors:** Xianzong Meng, Ping Chen, Andor Veltien, Tony Palavra, Sjors In't Veld, Joanes Grandjean, Judith R. Homberg

**Affiliations:** a Department of Cognitive Neuroscience, Donders Institute for Brain, Cognition, and Behaviour, Radboud University Medical Centre, 6525 AJ, Nijmegen, the Netherlands; b Department of Psychiatry, Radboud University Medical Centre, Nijmegen, the Netherlands; c Department of Medical Imaging, Radboud University Medical Centre, 6525 GA, Nijmegen, the Netherlands

**Keywords:** Neuroscience, Behavioral neuroscience, Methodologies, Approach-avoid conflicts

## Abstract

Animals need to respond to threats to avoid danger and approach rewards. In nature, these responses did not evolve alone but are always accompanied by motivational conflict. A semi-naturalistic threat imminence continuum model models the approach-avoidance conflict and is able to integrate multiple behaviors into a single paradigm. However, its comprehensive application is hampered by the lack of a detailed protocol and data about some fundamental factors including sex, age, and motivational level. Here, we modified a previously established paradigm measuring threat imminence continuum dynamics, involving modifications of training and testing protocols, and utilization of commercial materials combined with open science codes, making it easier to replicate. We demonstrate that foraging behavior is modulated by age, hunger level, and sex. This paradigm can be used to study foraging behaviors in animals in a more naturalistic manner with relevance to human approach-avoid conflicts and associated psychopathologies.

## Highlights

• We provide detailed guidance for setting up a modified paradigm for the threat imminence continuum model with commercial materials and open-source codes.
• We propose a modified paradigm for the threat imminence continuum model to quantify foraging behaviors.
• Our method enables comparison between groups as a function of multiple factors including age and food restriction.

## Graphical abstract

**Figure:**
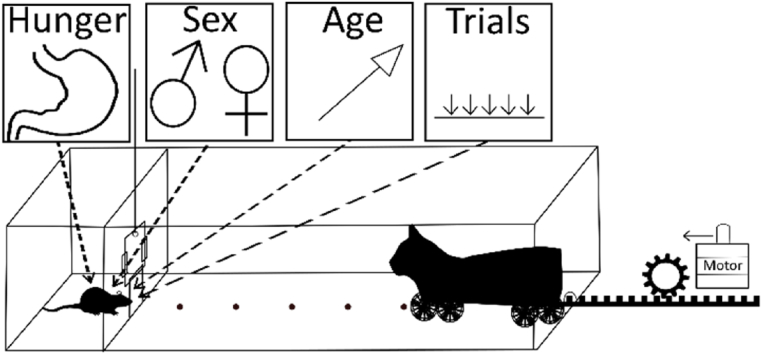

## 1 Introduction

Pleasure has been proposed to be evolution's boldest trick allowing animals and humans to seek primary rewards ensuring survival (Schultz, 2015). Importantly, the drive to seek and approach reward did not evolve in isolation but co-evolved with avoidance to prevent pain/punishment when threats are encountered. When encountering threats and rewards simultaneously, animals are compelled to choose between approach and avoidance behavioral responses (Amir et al., 2015; Choi and Kim, 2010; Mobbs et al., 2018; Hayden and Walton, 2014). Maladaptive approach-avoid conflicts have been implicated in the pathophysiology of major depressive disorder (Ironside et al., 2020), and are being used for the measurement of anxiety-related behaviors (La-Vu et al., 2020; Aupperle et al., 2023). Proper measurement of the approach-avoidance conflict as it occurs in real life is important for a fundamental understanding of survival behaviors, ecology, as well as human psychopathologies such as anxiety and depression (Rusconi et al., 2022; Kirlic et al., 2017). Therefore, a proper paradigm that could be used to investigate decisions during the foraging process of rats is needed.

Unfortunately, current animal paradigms cannot fully satisfy the measurement of the approach-avoidance conflict. Firstly, sometimes the conflict was measured separately (Hu, 2016). For example, in the fear conditioning test (Curzon et al., 2009), robust avoidance responses are triggered but we cannot assess the approach behaviors elicited by rewards. Conditioned suppression of lever pressing for food has been used for Pavlovian fear study including fear incubation (Pickens et al., 2010), fear learning and memory (McDannald and Galarce, 2011). While conditioned suppression of lever pressing is effective in capturing freeze behavior (McDannald and Galarce, 2011), it cannot measure the full range of behaviors involved. Our paradigm could help to overcome this limitation. Secondly, the difference between innate fear and learned fear was largely neglected (Corcoran and Quirk, 2007; Silva et al., 2016). Up to date, despite using different paradigms (e.g., Vogel, Geller-Seifter), most animal paradigms designed for approach-avoidance conflict studies are based on learned fear being associated with both reward and punishment. More importantly, these paradigms failed to address the conflict from a functional aspect. Animals in the wild have the opportunity to make all possible choices at any time. These behaviors can also influence each other (Fendt et al., 2020). This is not reflected in most current behavioral paradigms for rodents as the animals only get very limited choices. There is thus a clear need to provide alternative tests to examine behavior in animals across a range of dimensions with a strong translational value for associated studies in humans. Recently, several research groups have addressed this issue by devising novel approach-avoidance conflict tests in which foraging is the common assigned task, and food serves as reward (Engelke et al., 2021; Illescas-Huerta et al., 2021; Fernandez-Leon et al., 2021; Bravo-Rivera et al., 2021). The threat was generated by either foot shock (Illescas-Huerta et al., 2021; Fernandez-Leon et al., 2021; Bravo-Rivera et al., 2021) or a predator's odor (Engelke et al., 2021). A notable feature of these tests is the provision of a safe area. In these tasks rats were engaged in food foraging before threat exposure, thereby creating a conflict between food approach and threat avoidance behaviour. The multitude of potential paradigms makes it challenging to establish a “gold standard”. However, by standardizing specific settings and procedures, we could facilitate the replication of experiments, maintain the consistency of data between laboratories, and offer researchers a reliable starting point for fostering creativity and make versatile modifications for a broader range of research demands.

To better model approach-avoidance conflict as animals would display in the wild, we established a modified threat imminence continuum rat paradigm based on a previously published semi-naturalistic Robogator paradigm (Choi and Kim, 2010). The paradigm established by Dr. Jeansok Kim's lab has been used as a reliable method for fear conditioning (Zambetti et al., 2022), the integration of fearful information (Kong et al., 2021), sex effect on predator stimuli (Zambetti et al., 2019), and the neurophysiology of predator-induced fear (Kim et al., 2018). Here, we made some modifications to facilize its replication.

The paradigm consists of a safe nesting area and a foraging area. In the latter, food pellets are made available at various intervals. The threat is represented by a cat-shaped robot resting at the opposite end of the nesting area, propelling toward the rats according to pre-defined rules. By varying the distance, the rat needs to navigate to reach the pellet, we can modify the level of threat. This paradigm enables us to examine animal behavior under a more naturalistic condition while retaining the potential for consistency among laboratory experiments. Afterward, to allow a more reproducible implementation of this paradigm across laboratories, reproducible schematics are also provided.

Several modifications were made compared to the original paper we based our study on. Instead of a vague “mild restriction”, our food restriction regimen for the training process was meticulously calculated based on the rats' actual food consumption under free access conditions. Additionally, we modified the baseline days training from a variable range of 5–7 days to a fixed duration of 6 days. For the behavioral testing, instead of initially placing the food pellet in the middle and subsequently moving its location based on the rats' performance, we placed the food pellets in order from the nearest position to the nesting area to the farthest. Through this method, we could make the comparison of rats' performance at different positions, overcoming the limitation of solely focusing on the maximum distance rats could reach. As a result, we could detect more subtle behavioral changes. For instance, some manipulation may not change the maximum distance rats could reach, but can significantly alter the time required for rats to finish foraging at various positions. This contributes to a broader range of experimental possibilities. For statistics, we converted the time data from seconds into rank values, which improved the data's normality and facilitated its fit into a linear model.

Finally, we set out to investigate the behavioral consequences of important parameters for the optimization of the paradigm, namely sex, age, strain, and motivational levels. We find that sex, age and motivational levels affect rats’ foraging behaviors, and this paradigm can be applied to different rat strains.

## 2 Method

### 2.1 Preregistration, data and code availability

The statistical method is pre-registered (https://osf.io/v9ypz). The raw data are available under the term of the CC-BY license (https://doi.org/10.34973/38ba-f319, temporary reviewer access link: https://data.donders.ru.nl/login/reviewer-242207846/ZnQ2dnP1fgcjoUxVyvnv0TwQHnRNY0bJMcVnlOol1ec). The code and the processed data to reproduce the assay and the analysis are provided under the terms of the Apache-2.0 license (https://gitlab.socsci.ru.nl/preclinical-neuroimaging/robotmod).

### 2.2 Subjects

All procedures were carried out in agreement with the current National Research Council Guide for the Care and Use of Laboratory Animals and were approved by the central commission for animal experiments (CCD) in The Hague (the Netherlands), license number AVD10300 2020 9185. All efforts were made to reduce the number of animals used and their suffering. We used mixed-sex groups with a 1:1 sex ratio. Specifically, 12 male and 12 female Sprague Dawley (SD) rats, and 12 male and 12 female Long-Evans (LE) rats were purchased from Charles River (Calco, Italy). For SD rats, two batches of animals were used to test the effect of age on the approach-avoidance conflict in the paradigm. We used 12 postnatal day (PND) 70 rats initially weighing 420–500 g (6 males) and 270–320 g (6 females) and 12 PND 140 rats initially weighing 560–660 g (6 males) and 300–350 g (6 females). For LE rats, we further tested the effect of food restriction on the approach-avoidance conflict. We used 24 PND70 rats initially weighing 360–450 g (12 males) and 220–270 g (12 females). Upon arrival, animals were given two weeks for acclimation to the reversed day-light cycle with free access to food and water. The body weight after this acclimation is considered as initial body weight. Animals were housed in groups of 2 in Macrolon type III cages (42 × 26 × 15 cm) under a 12 h/12 h reversed day/night cycle (lights off at 8:00 a.m.) in a temperature-controlled room (22 ± 2 °C). Experiments were performed during the dark phase. To motivate the animals for the test, they were subjected to a food restriction regime with *ad libitum* water to maintain 85–95% of their normal weight.

### 2.3 Threat imminence continuum model setup

The apparatus was assembled by Plexiglas (methyl methacrylate) plates and enhanced by L-shaped anchor plates from the outside with industrial glue (Supplementary Fig. 1A). Afterward, another plate serving as a separating plate was positioned inside to create a nesting area (29.21 cm length × 57.12 cm width × 59.69 cm height) and a foraging area (201.93 cm length × 58.42 cm width × 60.96 cm height). On the separating plate, a 10 cm × 10 cm area was laser-cutted at the center of the bottom part, and a remotely controlled door was built to open or close the access from nesting area to foraging area (Supplementary Fig. 1B). The robot was attached to a gear rack and the movement was driven by a NEMA17 step motor controlled by an Arduino system. A Raspberry Pi Night Vision Camera (Product code WS-10299) was placed above the center of the cage to record the video (30 frames/s) from both the nesting and foraging areas. All the behavioral tests were performed in a dark experimental room with red light. A customized LEGO-based Robot (66.04 cm length, 17.78 cm width, 15.24 cm height) as shown in Supplementary Figs. 1C–1E, was coded to surge 23 cm (at a velocity of ∼ 75 cm/s) and return to its starting position via an Arduino system (Supplementary Fig. 2).

### 2.4 Food restriction regime

Food consumption was measured under a free feeding condition for a week. Afterward, animals received less food based on the calculation from free-feeding conditions throughout the whole experiment. For instance, 90% food restriction means animals could only get 90% of the food they consumed under free access. That is, if the rats could eat 40g food per day under free access conditions, they received 36g food per day to achieve 90% food restriction. During behavioral procedures, food was provided after training or testing. Rats were maintained at 85–95% of their body weight.

### 2.5 Behavioral procedures

Rats underwent successive stages of habituation days, baseline days, and robot encounter days. The procedure is demonstrated in Supplementary Fig. 3.

#### 2.5.1 Habituation days

Animals were placed in the nesting area for 30 min/d for 3 consecutive days with three food pellets (grain-based, 0.8–1.3 g) inside to acclimatize to the nesting area, during habituation days. The door was closed and animals cannot enter the foraging area.

#### 2.5.2 Baseline days

During baseline days, the Robot was absent. After a minute in the nesting area (no food pellets), the door to the foraging area would be opened, and the animal was allowed to explore and search for a food pellet placed 25.4 cm from the nest area (first trial) for 5 min. As soon as the animal took the pellet back inside the nest, the gateway closed. Once the animal finished consuming the pellet, the second foraging trial (with the pellet placed 50.8 cm from the nest area) and then the third foraging trial (with the pellet placed 76.2 cm from the next area) started in the same manner. Animals underwent 6 consecutive baseline days. Data from all trials were included in the data analysis, even if the rat failed to collect the food pellet. The trial performance was converted into a score value of 7 according to the Rank-score conversion Table (Supplementary Table 1).

#### 2.5.3 Robot encounter day

On the robot testing day, the Robot was placed at the opposite end of the foraging area. The food pellet was placed at 25.4 cm, 50.8 cm, 76.2 cm, 101.6 cm, and 127 cm locations in order. After opening the door, each time the animal approached ∼1–3 cm from the pellet with its face directed towards the food, the Robot was manually triggered, surged 23 cm toward the pellet and returned to its original position. Animals were permitted 3 min to procure the pellet. If animals could procure the food pellet, the door would be closed when animals returned to the nesting area with a food pellet. Subsequently, the food pellet would be placed in the next position. If animals could not procure the food pellet, the door would close with the animal inside the nesting area after 3 min. Under this condition the food pellet was also placed in the next position. Data from all trials were included in the data analysis even if the rat failed to collect the food pellet. The trial performance was converted into a score value of 7 according to the Rank-score conversion Table (Supplementary Table 1).

The latency time required for animals to take the food pellet back to the nesting area, the latency required for the animal to go outside of the nesting area, the latency required for animals to approach to food pellet for the first time, and for each position, the number of animals could procure the pellet successfully served as the dependent variables.

### 2.6 Statistical analyses

Statistical analysis was optimized on data robot testing day 1 in SD rats. Analysis was performed using R (Version 4.1.2) using the lme4 (lme4_1.1–27.1), multicomp (multcomp_1.4–18), effect size (effectsize_0.6.0.1), and performance (performance_0.8.0) packages. A linear mixed model was built with sex, trials, sex:trials interaction effect, and, if relevant age or food restriction, as fixed effects, and interaction effect with sex or trials, individual intercepts as random effects using the following model: lmer (measured.variable ∼ age or food restriction + sex + trials + sex:trials + age or food restriction:sex + age or food restriction:trials + (1|ratID)). As a prerequisite to hypothesis testing, the assumptions of normality of the residuals were tested using the performance package. We applied a rank-score conversion (Supplementary Table 1) to enhance the normality of the residuals and minimize the Akaike information criterion (AIC) and Bayesian information criterion (BIC) scores (comparing raw score vs. rank-score, AIC = 785.2 vs 484.8; BIC = 813.6 vs 518.3).

Interpretations of effect size follow Cohen's 1998 guidelines (Cohen, 1988), namely small effect: η2 > 0.02; medium effect: η2 > 0.13; large effect: η2 > 0.26. Results of effect size were presented as effect size (eta-squared, η2) and the 95% confidence intervals (eta-squared upper confidence interval is set to 1 by default).

Contrast analysis at individual positions was done by R using the model based package (modelbased_0.8.6). t and df values were converted to Cohen's d values. The interpretation of the effect sizes follows Cohen's 1998 guidelines (Cohen, 1988), namely small effect: d > 0.2; medium effect: d > 0.5; large effect: d > 0.8.

## 3 Result

### 3.1 Younger rats finish foraging more rapidly

**Fig. 1:**
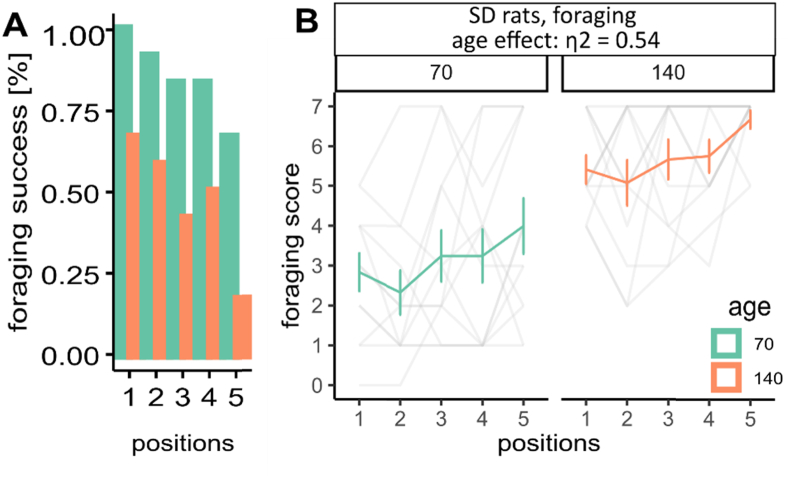
Age effect on rats' foraging behavior. (A) success rate of younger (PND 70) and older (PND 140) rats in different sex. (B) time to finish foraging. Age effect η2 = 0.54, [0.28, 1.00], n_PND 70_ = 12 (6/6 (m)male/(f)female), n_PND 140_ = 12 (6/6 m/f). Error bars indicate ± 1 standard deviation.

The foraging behaviors change across the life span in both human beings and rodents (Clark, 1980; Mata et al., 2013; Shoji and Miyakawa, 2019; Boguszewski and Zagrodzka, 2002). Firstly we wanted to test the effects of age on animals’ foraging behaviors in the threat imminence continuum model. Two batches of SD rats at different ages (PND 70 and PND 140, n = 12 each, 6/6 m/f) under 90% food restriction were used. We define the success as rats who could successfully bring the food pellet back to the nesting area within the allocated time. Using this metric, we find that older animals had an substantial lower rate of success than younger animals (e.g. PND70 vs PND140 at position 1: 91.7% vs 66.7%; Fig. 1A). Next, we tested the overall time needed to reach the food and return to the nesting area, henceforth referred to as foraging. In SD rats data (n = 24, including both PND 70 and PND 140 group), we found that using raw values in seconds broke assumptions of normality of the residuals due to bounded values or missing data in failed trials. To overcome this, we converted raw values to scores (Supplementary Table 1). After correction, we found that older animals needed more time to procure the food pellets relative to younger animals (Fig. 1B, age effect: η2 = 0.54, [0.28, 1.00]; trial effect η2 = 0.16, [0.04, 1.00]) at all positions (Supplementary Table 2). The position effect denotes longer times to bring pellets back to the nesting areas as a function of the pellet distance from the nest, and will not be discussed further in the text as this is an expected effect in this paradigm. From this experiment, we conclude that younger rats (PND 70) might be more suitable for testing because they avoid a floor effect caused by insufficient success rate, while providing sufficient margins for either improvements or deteriorations in experimental groups.

Since age in rats correlates with body weight, the independence of age from weight was assessed. To this end, we explored the effect of body weight on foraging behaviors. A small age and weight interaction effect was found in the SD dataset (η^2^ = 0.04, [0.00, 1.00]), suggesting that the age effect is independent of body weight. Regarding the weight effect, a medium effect on rats’ foraging was found among SD rats (η2 = 0.16, [0.00, 1.00]). However, the weight effect was small for LE rats (η2 = 0.01, [0.00, 1.00]). We hypothesize that the medium weight effect among SD rats is likely due to the mild food restriction regime.

### 3.2 Hunger modulates foraging behavior

**Fig. 2:**
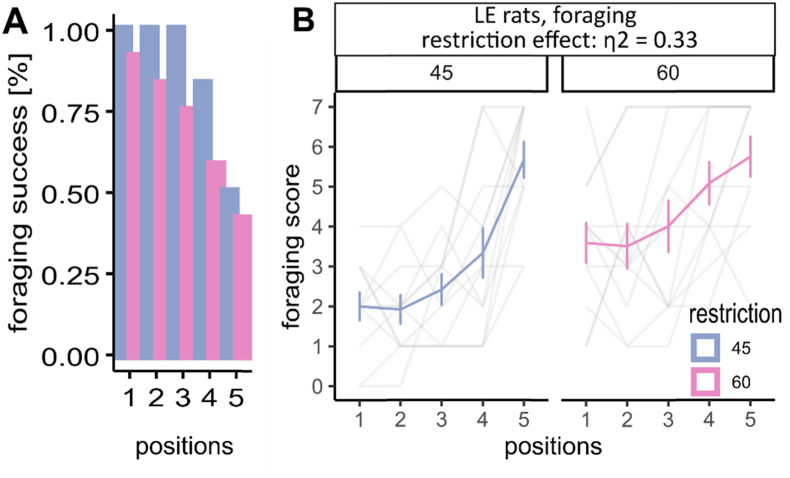
Food restriction effect on rats' foraging behavior. (A) success rate of foraging under different restrictions in different sexes. (B) time to finish foraging. Food restriction effect η2 = 0.33, [0.07, 1.00], n_45%_ = 12, n_60%_ = 12. Standard deviations are indicated as the vertical error bars.

In behavioral studies, food restriction is routinely used to initiate or maintain motivational status for animals to engage in different tasks (Dietze et al., 2016), as well as to enhance the motivation to forage for food (Burnett et al., 2016). In our foraging task, a proper food restriction is necessary. However, the extent of food restriction likely influences the foraging behaviors including approach-avoidance decision-making, foraging strategies, speed, hoarding activity, etc. (Hernández et al., 2019; Gutman et al., 2007; Heinz et al., 2021; Burnett et al., 2016; Dailey and Bartness, 2010; Keen-Rhinehart et al., 2010). Here, we set out to investigate the effect of food restriction. We put LE rats under either 45% (n = 12, male:female ratio = 1:1) or 60% (n = 12, male:female ratio = 1:1) food restriction for 7 days before training and testing the animals with the Robot assay. In this study, we implemented 45% or 60% food restriction regime for rats, which entailed providing rats with either 45% or 60% of the food they consumed under free feeding conditions, thus 45% restriction is associated with a higher hunger level. We find that the success rate increased under the stricter restriction (e.g. 45% restriction vs 60% restriction at position 1 & 5: 100% vs 91.7%; 50% vs 41.7%; Fig. 2A). Meanwhile, food restriction has a large effect on foraging performance (restriction effect: η2 = 0.33, [0.07, 1.00]; trial effect: η2 = 0.50, [0.37, 1.00]; sex effect: η2 = 0.33, [0.07, 1.00]), as animals under a 45% restriction regime could take food pellets quicker than animals under 60% restriction regime (Fig. 2B). The more hungry the rats were (45% restriction vs 60% restriction), the quicker they were during foraging, except when the food pellet was extremely close to the Robot. In other words, when the threat level was extremely high, the food restriction had limited effect on rats’ foraging behavior (Fig. 2B, Supplementary Table 3).

### 3.3 Are female or male rats faster to finish foraging?

**Fig. 3:**
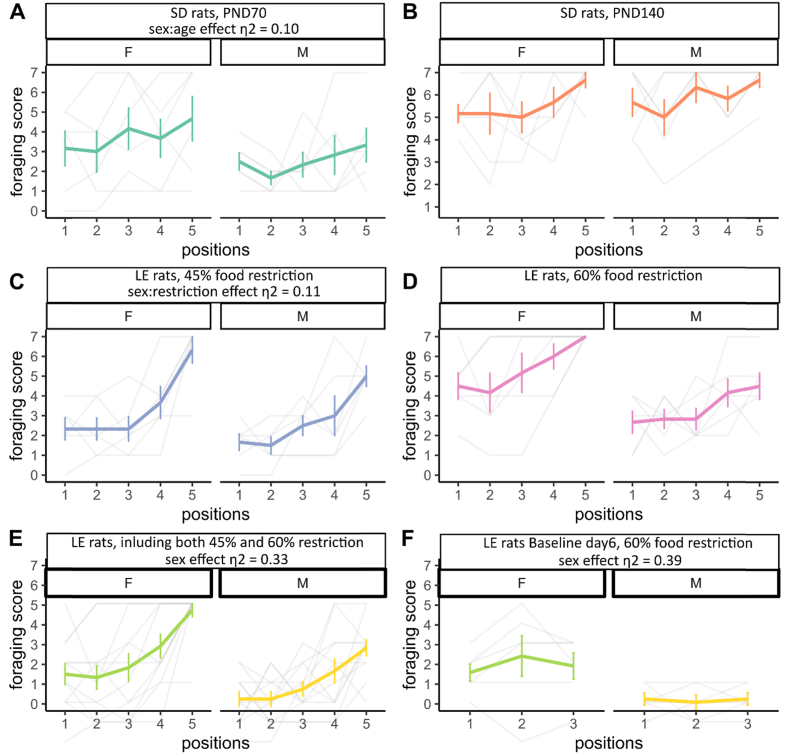
Sex effect on rats' foraging behavior. (A) Time to finish foraging of SD rats at PND70. (B) Time to finish foraging of SD rats at PND140. Sex:age effect η2 = 0.10, [0.00, 1.00], n_PND 70_ = 12, n_PND 140_ = 12. (C) Time to finish foraging of LE rats under 45% food restrictions. (D) Time to finish foraging of LE rats under 60% food restrictions. Sex:restriction effect η2 = 0.11, [0.00, 1.00], n_45%_ = 12, n_60%_ = 12. (E) general comparison between males and females of time to finish foraging. Sex effect η2 = 0.33, [0.07, 1.00], n_male_ = 24 (12/12 45%/60%), n_female_ = 24 (12/12 45%/60%). (F) general comparison between males and females of time to finish foraging at Baseline day 6. Sex effect η2 = 0.39, [0.03, 1.00], n_male_ = 24 (12/12 45%/60%), n_female_ = 24 (12/12 45%/60%). Standard deviations are indicated as the vertical error bars.

As a large sex effect was found from the above food restriction study, we aimed to investigate the effect of sex on test performance. Sex differences in risky-foraging behavior have also been reported when facing aerial predator stimuli. Specifically, females exhibited a stronger fear response than males, which was characterized by a longer latency to finish foraging regardless of the presence of the threat, a slower habituation to threat, and a stronger contextual fear response after the removal of threat (Zambetti et al., 2019). In line with the aerial predator study, sex had a larger effect (η2 = 0.33, [0.07, 1.00]) on rats’ foraging performance in the food restriction effect study. However, in our age effect study, only a small sex effect (η2 = 0.03, [0.00, 1.00]) was detected. This may be due to the mild food restriction regime. Meanwhile, the small sex:age interaction effect indicates that age is not a function of sex differences in our paradigm (Fig. 3A and B).

At the same time, the small sex:restriction interaction effect suggests that the sex effect is independent from food restrictions. (Fig. 3C and D). To further investigate the sex effect, we pooled the data from the LE rats together (data from both food restriction regimes), and imported data from Baseline day 6 (the last day of Baseline days). We noticed that female rats needed more time to complete foraging (Fig. 3E), even when the robot was not presented (η2 = 0.39, [0.03, 1.00]) (Fig. 3F).

### 3.4 Probing decision-making and risk assessment with leaving and approaching behaviors

The food foraging paradigm plays an important role in behavioral science, and besides foraging behavior itself, the paradigm also involves higher cognitive functions such as decision making and risk assessment. The paradigm we present here allows researchers to measure these advanced behaviors. First, the time the animals need to leave the nesting area (leaving behavior) could reflect animals' decision making process between starvation risk and predation risk. Secondly, after leaving the nesting area, the time animals need to approach the food pellet for the first time (approaching behavior), could reflect animals’ foraging strategies to prevent or defer the progression of threat.

**Fig. 4:**
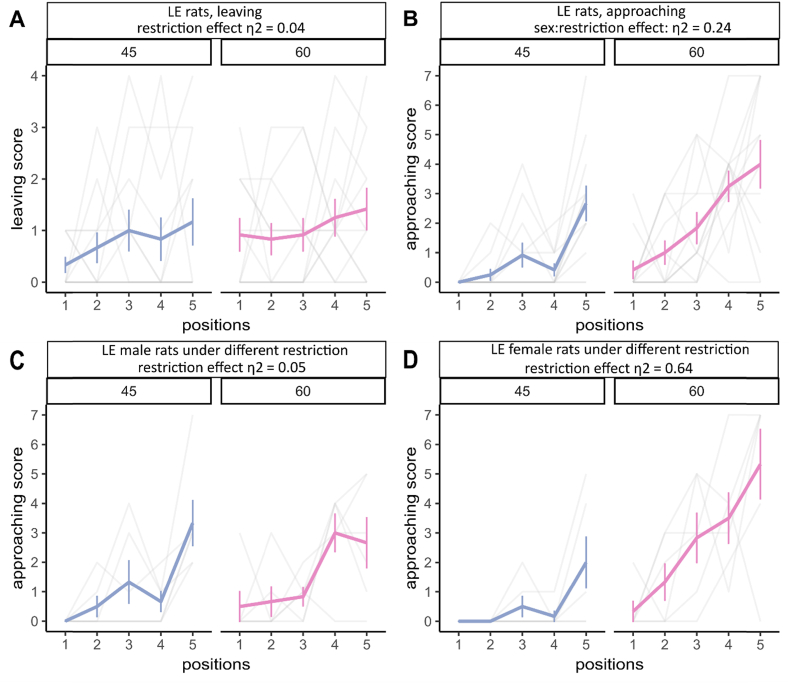
Leaving and approaching behavior during foraging. (A) time to leave the nesting area. Restriction effect η2 = 0.04, [0.00, 1.00]. (B) time to approach the food pellet for the first time. Sex:restriction effect η2 = 0.24, [0.02, 1.00]. (C) Approaching score for LE male rats under different food restriction conditions. Restriction effect η2 = 0.05, [0.00, 1.00]. (D) Approaching score for LE female rats under different food restriction conditions. Restriction effect η2 = 0.64, [0.26, 1.00]. n_45%_ = 12, n_60%_ = 12. Standard deviations are indicated as the vertical error bars.

Meanwhile, these behaviors in foraging could be regulated by animal's internal state, e.g. satiety state. Therefore, we tested the leaving behavior and approaching behavior under different food restriction regimes. For leaving behavior, no obvious deviations were observed (Fig. 4A) (food restriction effect η2 = 0.04, [0.00, 1.00], sex effect η2 = 0.04, [0.00, 1.00] and trial effect η2 = 0.07, [0.00, 1.00]). For approaching behavior, a medium sex:restriction effect (η2 = 0.24, [0.02, 1.00]) was observed, indicating that the sex effect and food restriction effect were dependent on each other. Specifically, sex has a medium effect (η2 = 0.18, [0.00, 1.00]) under the 45% restriction condition, and a large effect (η2 = 0.29, [0.00, 1.00]) under the 60% restriction condition (Fig. 4B). Meanwhile, restriction has a small effect (η2 = 0.05, [0.00, 1.00]) on males (Fig. 4C) but a large effect (η2 = 0.64, [0.26, 1.00]) on females (Fig. 4D).

## 4 Discussion

Here, we established a modified paradigm based on the threat imminence continuum model to quantify foraging behaviors. Using this paradigm, the effects from some crucial animal parameters including age, sex and food restriction on the foraging behaviors were investigated. Specifically, age and food restriction affected the success rate. We also found that sex influenced performance, however, this seems to be independent of the presence of a threat. We propose that using younger animals with a moderate food restriction regime helps to avoid flooring or ceiling effects, thus allowing the detection of a larger dynamic range of behavior. Finally, to foster further research we provide schematics, code, and assembly instructions to enhance the replication of this modified paradigm.

We found that younger rats could finish foraging faster than older rats. Rats at the age of 2 months are considered as adults because of the maturation of vital systems (Sengupta, 2013; Andreollo et al., 2012; Quinn, 2005; Klein and Romeo, 2013). Social maturity starts at the age of 5 and 6 months (Sengupta, 2013; Andreollo et al., 2012). Although most studies categorized all rats from 2 to 5 months as adult rats, the decrease of mobility in adulthood cannot be overlooked (Boguszewski and Zagrodzka, 2002; Sudakov et al., 2021; Arrant et al., 2013). Meanwhile, clear age-dependent foraging differences have also been reported in rats before, including foraging strategies, food preference and tolerance etc. (Clark, 1980; Bert et al., 2006). This suggests that the foraging difference we observed was not solely caused by the different locomotion between younger and older rats, but also by a difference in approach-avoidance decision-making. The success rate of younger rats was always higher than older rats in both sexes, indicating that younger rats were more willing to take the risk. However, as to whether the lower success rate in older rats is attributable to reduced motivation for food, changes in punishment sensitivity, or impaired cognitive flexibility still needs to be validated by further experiments.

Food restriction is a common tool used to motivate animals for motivated behaviors of hunger, or learned behaviors (Dietze et al., 2016; Bert et al., 2006; Carlini et al., 2008; Conrad, 2010; Kant et al., 1988; Heiderstadt et al., 2000). One alternative method is using palatable rewards (Toth and Gardiner, 2000; Young et al., 1955; Carroll et al., 1989; Ray, 1998). However, the latter might not generate the level of performance as obtained by food restriction. Palatable food can motivate animals to perform without food deprivation in some circumstances, especially if the task is simple for animals. However, it might take longer for sated animals to learn the task than deprived animals. Sometimes sated animals learn poorly or even refuse to perform the task (Kant et al., 1988; Toth and Gardiner, 2000; Hughes et al., 1994; Collier and Levitsky, 1967; Lattal and Williams, 1997). Considering that rats would be foraging in a fearful environment under predation risk, we believe that food restriction could induce a more reliable performance. During the Robot testing process, rats are given 3 min to procure the food pellet from each position. Thus most ideally rats could get food pellets within 3 min after baseline days training. Under 90% food restriction conditions, on baseline day 6 (the last day of baseline training), younger male rats were all able to finish foraging within 3 min. However, all older male rats failed to pass this criterion. Meanwhile, for females, the rates were 66.7% and 16.7% in the younger group and older group, respectively. More importantly, the sex effect in the older group was very small, which is in conflict with reports that female rats are more reluctant to enter a large, novel, open foraging area to procure the food pellets (Zambetti et al., 2019; Pellman et al., 2017; Carrier and Kabbaj, 2013; Jolles et al., 2015; Turner and Burne, 2014). We noticed that under this very mild restriction regime, rats maintained a stable body weight and sometimes even gained more weight. This observation raised our consideration regarding the representativeness of our experimental conditions compared to real-world scenarios. Meanwhile, as shown in Fig. 1B, foraging performance across different positions was found to occur within a narrow dynamic range (foraging score 3–4). We believe that the main reason for this phenomenon is the insufficient food restriction. Because both male and female rats were not motivated enough, many rats refused to go foraging in the risky environment, compromising the sex effect. To improve the sensitivity of our paradigm, we tested two other food restriction regimes, 45% and 60%. We also used a more active rat strain, Long Evans. Data revealed that the success rate gradually reduced along with the distance away from the nesting area as anticipated, and that the more hungry the rats were, the quicker they could finish foraging, except when the food pellet was extremely close to the Robot, or in other words, when the threat level was extremely high. It also should be noted that rats under the 45% food restriction could forage very quickly especially from the first four locations in an average of 12–35 s, which may lead to a ceiling effect. Based on the results above, we recommend using the food restriction at a range around 60%.

As for the sex differences in foraging, when facing the threat, our results indicate that females, in general, take a longer time to finish foraging. This finding is consistent with a previous report that female rats exhibited more potent fear responses to aerial threats (Zambetti et al., 2019). However, this phenomenon seems to be threat-independent (even when the Robot is not present). Therefore, we further would like to conclude that our assay may not be a very robust tool to tweak out sex differences because of the fundamental sex differences on rats’ foraging behaviors. Meanwhile, it also has been reported that male and female rats would use different strategies when the threat was integrated into their life. That is, male rats would be more willing to put effort in risky foraging because it can increase mate access (Ellis et al., 2009; West-Eberhard, 2014), whereas female rats would slash their requirement of food in order to avoid aversive stimuli (Pellman et al., 2017). However, whether it is due to the alteration of fear perception or adaptation of strategy still needs to be investigated.

The effect from above mentioned factors on rats' leaving and approaching behaviors was also investigated, which enables us to explore more cognitive functions, including risky decision making and risk assessment. When the door was opened, animals would be able to see the Robot and were required to make a decision whether or not to leave the nesting area for foraging. As proposed by Fendt et al. (2020), the decision-making of animals is based on the integration of various factors like hunger, distance of threat, etc. In the present study, two internal factors, hunger level (45% and 60%) and sex, and one external factor distance of threat was studied. Results indicate that all above mentioned factors only have a small effect on rats’ foraging decision. Interestingly, it has been previously reported that males exhibit greater risk-taking than females in decision making when involving the risk of punishment (Ellis et al., 2009). However, in our paradigm, sex only exhibited a small effect on foraging decision (leaving behavior). Therefore, we cannot exclude the possibility that once the hunger level has reached a certain threshold, the effect from sex and distance of threat on the decision-making process will have a very limited impact. Thereby, optimizing the food restriction regime is highly recommended when using this paradigm for decision-making studies. For approach behavior, as reported by Kim et al. in their paper using a similar paradigm (Choi and Kim, 2010), with the appearance of Robot, rats do not simply give up foraging, but try to use different strategies and repeated efforts to procure the food pellets. This suggests the involvement of risk assessment. Results revealed a medium sex:restriction interaction effect, and further analysis found that under a milder restriction regime, sex could have a larger effect on the risk assessment process. Furthermore, the results suggest that food restriction has a more profound effect on females compared with males. The mechanism behind this phenomenon still needs deeper investigation. However, it should be noted that ovariectomy in adult female rats did not alter the risky foraging strategies, implying that these changes are prepared during early development (Pellman et al., 2017). Here, we assume that the difference in threat perception, information integration, and ghrelin secretion between males and females might contribute to the changes. Yet, this also still needs to be further investigated.

Defensive behaviors strengthen survival and enhance animals' reproductive rate or capacity (Williams, 1991). The “threat imminence continuum” theory (Fanselow and Lester, 2013; Bouton et al., 2001) postulates that animals' response escalate along with three phases, spanning from pre-encounter phase to circa-strike phase: during the *pre-encounter phase,* the threat still has not been detected yet, however, when the likelihood of predation increases or a higher predatory imminence animal perceived, the exploration and foraging behaviors will be reduced (Fanselow and Lester, 2013); during *post-encounter phase*, freezing is the dominant defense response, which often elicits stress-enhanced fear learning (Hoffman et al., 2022). Finally, during *circa-strike*, animals will have direct contact with the threat, which elicits an outburst of threat avoidance or combat. In this paper, we did not directly measure the defensive behaviors, however, our paradigm allows the recording of the animals' performance during experimental sessions, and through tools like EthoVision or DeepLabCut, the various defensive responses exhibited by the animals, including freezing, flight, and fight, can be quantified and analyzed. To enhance the distinction of behaviors, we recommend using multiple cameras from different angles. Moreover, our open-source data can facilitate the analysis of additional parameters. Through animal tracking tools other measurements could be derived from the data including the rat's velocities, trajectories, number of foraging attempts etc. We encourage researchers to be creative to make this paradigm more versatile and able to cover a broader range of research demand.

Some limitations should be taken into account when interpreting the data. Firstly, the different visual acuities between males and females might also account for the observed sex differences (Seymoure and Juraska, 1997; Juraska, 2022). Secondly, after 6 days of baseline training, the possibility cannot be ruled out that rats decided to leave the nesting area too quickly to clearly see or notice the appearance of the Robot. Therefore, for experiments focusing on foraging decision-making processes, we recommend replacing the separating plate with a transparent one, and triggering the Robot a few times before behavioral testing to allow rats to perceive the threat in advance. Thirdly, in this study, the risk assessment was evaluated by measuring the time it took for rats to attempt to procure the food pellet for the very first time after leaving their nesting area. This method for evaluating risk assessment is simple and direct, however, it should not be overlooked that approaching to food behavior is regulated by some other factors, e.g. social cues (Hall et al., 1997). Therefore, the specificity of the risk assessment measure might need to be improved, which could be achieved by analyzing specific behaviors of the rats, including head-dipping, rearing, grooming, etc., especially Stretch-attend posture, which is currently the most prevalent method (Holly et al., 2016; Albrechet-Souza et al., 2007; Magara et al., 2015; Coimbra et al., 2017; Rodgers et al., 1999; Reis et al., 2012). Furthermore, in this paradigm, the food is provided after training or behavioral testing, that makes our paradigm an “open economy” type, which narrows the detection range of behaviors (Pellman and Kim, 2016) and excludes information from temporal aspects of fear and anxiety (Schuessler et al., 2020). As proposed by Collier et al. (1972) a “closed economy” paradigm in which animals obtain their daily food exclusively from operant processes and typically live in the operant chambers could provide be a more holistic approach to for rodent behavior study. By introducing aversive components to the “closed economy”, “risky closed economy” was developed (Fanselow et al., 1988; Helmstetter and Fanselow, 1993), through which the information from a spatiotemporal dimension of fear and anxiety could be obtained. And its naturalistic qualities could facilitate the assessment of fear and anxiety in decision-making (Mobbs et al., 2018). However, some practical limitations of “Risky Closed Economy” might hinder its application, e.g. it requires substantial amount of time and space and rats' social interaction may be reduced because animals need to be single housed (Schuessler et al., 2020). In our experiments, when applying 45% food restriction, body weight of the rats dropped approximately by 10–15% over the food restriction week. To mitigate discomfort of the rats without reaching a human endpoint, 45% food restriction was set as the most stringent regime. However, a wider food restriction range could reveal more subtle patterns in foraging behaviors. Besides, measuring stress hormones like corticosterone could provide a fuller picture of rats’ fear level as well as validate how much fear the robot triggered (Lesuis et al., 2018; Korte, 2001).

In conclusion, we propose a modified paradigm based on the threat imminence continuum model which could enable us to quantify foraging behaviors under threat. Some key factors influencing foraging and approach-avoidance behavior were investigated. Results suggest that age, sex and hunger level affect rat's foraging performance, as well as risk assessment. However, whether these factors could also affect risky decision-making still needs to be further investigated. Besides, our paradigm could serve as a powerful tool to exploit the full potential of comprehensive behavioral phenotyping of rats' foraging under threat in a semi-naturalistic environment.

## Credit author statement

J.H. and J.G. design and supervision. X.M., A.V. experimental cage building. X.M., T.P. data collection, X.M., J.G., P.C., data analysis. X.M., S.V. Python coding. J.H., J.G., X.M., P.C., A.V. paper writing and editing. X.M. was the consistent human experimenter who was present in the experiment room and primarily responsible for interacting with the subjects during the entire duration of the study. All authors had full access to all of the data in the study and accept responsibility for the decision to submit for publication.

## Declaration of competing interest

The authors declare no competing interests.
