# Supplementary material for: Estimating foraging behavior in rodents using a modified paradigm measuring threat imminence dynamics
Source: Neurobiol Stress. 2023 Nov 7;28:100585. doi: 10.1016/j.ynstr.2023.100585 (PMC10661863; doi:10.1016/j.ynstr.2023.100585)
Supplement: Multimedia component 1 [file mmc1.docx]

**Supplementary information**


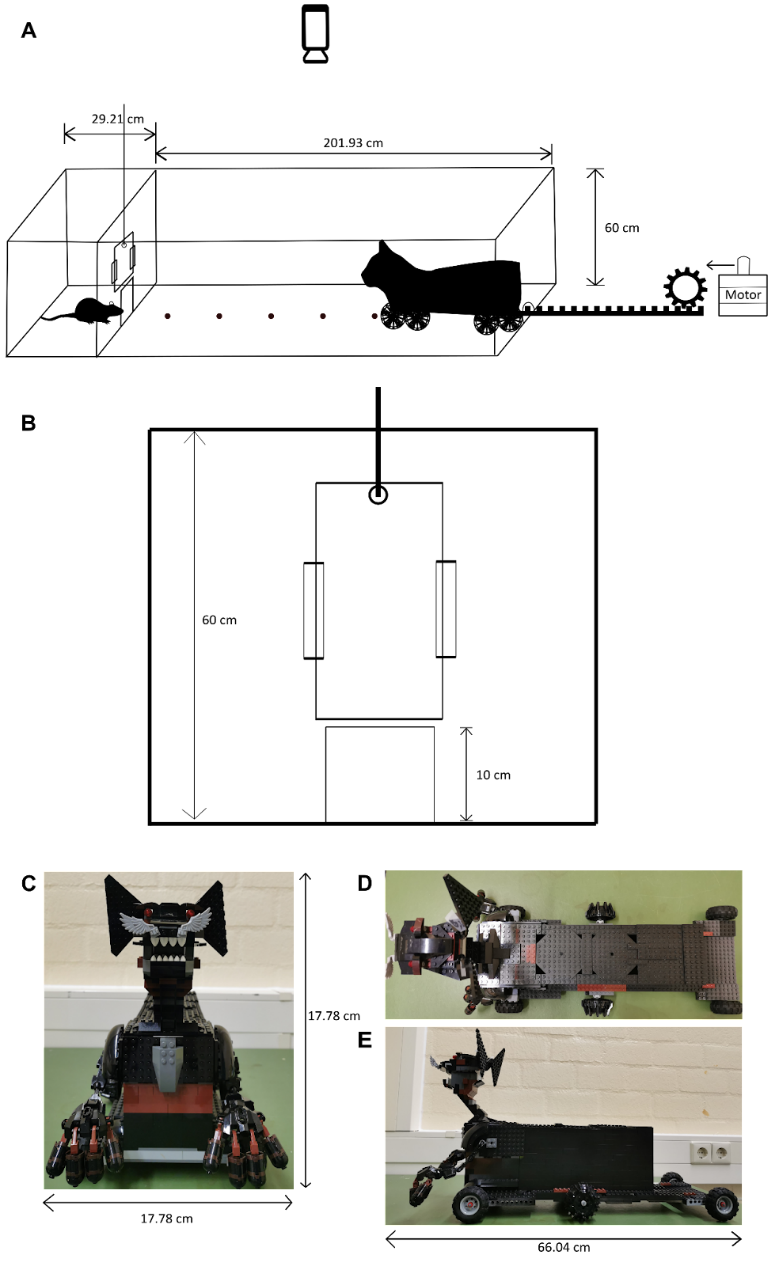


Supplementary Fig 1. Schematics of the paradigm setup. (A) Overview of the paradigm. (B) Controlled-door on the separating plate. (C) The front view of the Robot. (D) The top view of the Robot. (E) The side view of the Robot. Note: for building the setup, we recommend controlling the door manually to avoid causing injury and extra stress to rats, because rats always poke their head out before going foraging and sometimes leave their tails outside the nesting area when taking food back. During the experiments, it was observed that rats often made multiple attempts to obtain the pellet, and that the pellet's position could be moved with each attempt. Therefore, the Robot was triggered manually. Nonetheless, the automated robot trigger system implemented in our code could be used by researchers who wish to use a reward that can be fixed in position, such as juice or a sweetened water fountain for the rat.


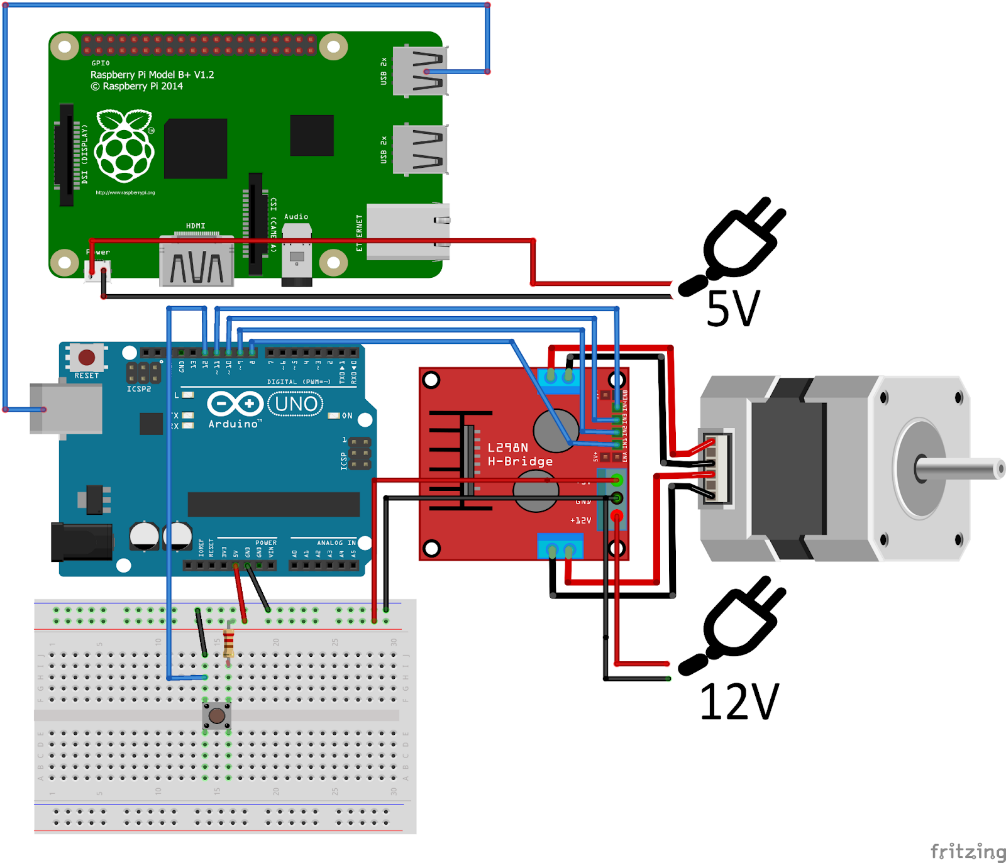


Supplementary Fig 2. Schematics of connections between the Arduino board, Raspberry Pi and Step motor.


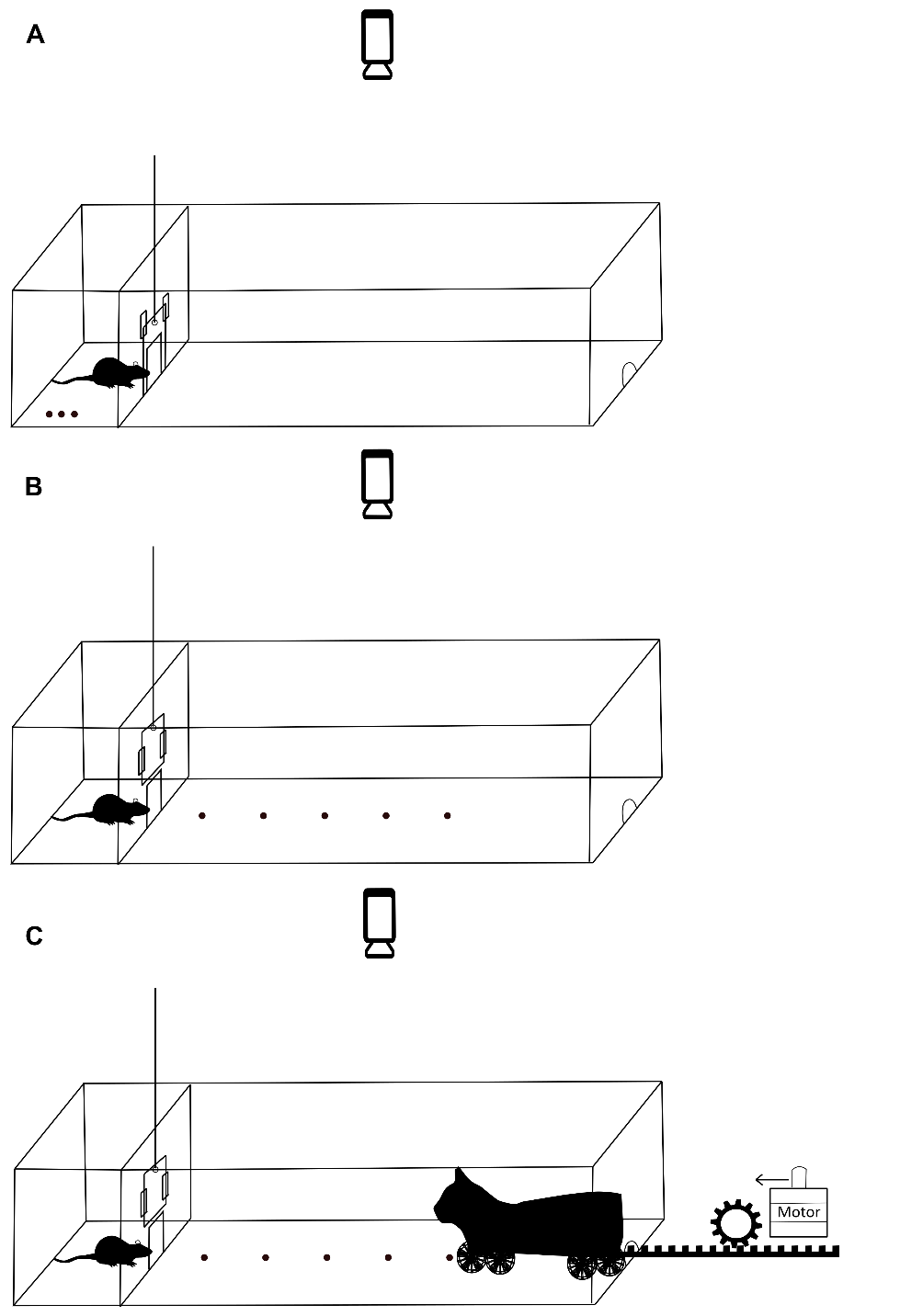


Supplementary Fig 3. Schematics of behavioral procedures. (A) Habituation. (B) Baseline days. (C) Robot testing day.

Supplementary Table 1. Rank-score conversion

| Animals’ performance | score value |
| --- | --- |
| failed to complete the task | 7 |
| complete the task using more than 200 sec | 6 |
| complete the task within 200 - 100 sec | 5 |
| complete the task within 100 - 50 sec | 4 |
| complete the task within 50 - 25 sec | 3 |
| complete the task within 25 - 12.5 sec | 2 |
| complete the task within 12.5 - 6.25 sec | 1 |
| complete the task within 6.25 - 0 sec | 0 |

Supplementary Table 2. Age effect on rats’ foraging behavior at different positions.

| Positions | 1 | 2 | 3 | 4 | 5 |
| --- | --- | --- | --- | --- | --- |
| Cohen’s d value | 0.86 | 0.92 | 0.81 | 0.83 | -0.89 |
| 95% confidence interval | [-1.37, -0.35] | [-1.43, -0.40] | [-1.31, -0.30] | [-1.34, -0.32] | [-1.40, 0.37] |

Supplementary Table 3. Food restriction effect on rats’ foraging behavior at different positions.

| Positions | 1 | 2 | 3 | 4 | 5 |
| --- | --- | --- | --- | --- | --- |
| Cohen’s d value | 0.56 | 0.56 | 0.56 | 0.62 | -0.03 |
| 95% confidence interval | [-1.01, -0.10] | [-1.01, -0.10] | [-1.01, -0.10] | [-1.07, -0.16] | [-0.48, 0.42] |

| A.  |
| --- |
| B.  |

Supplementary videos. (A). Representative video for baseline days, food pellet was placed at position 3. (B) Representative video for Robot testing days, food pellet was placed at position 3. Note: the video has been speedup for demonstration purpose.
